# Ionizing Radiation Increases the Activity of Exosomal Secretory Pathway in MCF-7 Human Breast Cancer Cells: A Possible Way to Communicate Resistance against Radiotherapy

**DOI:** 10.3390/ijms20153649

**Published:** 2019-07-25

**Authors:** Nasrollah Jabbari, Muhammad Nawaz, Jafar Rezaie

**Affiliations:** 1 Solid Tumor Research Center, Cellular and Molecular Medicine Institute, Urmia University of Medical Sciences, Urmia 57591, Iran; 2 Department of Rheumatology and Inflammation Research, Institute of Medicine, Sahlgrenska Academy, University of Gothenburg, 41346 Gothenburg, Sweden

**Keywords:** exosomes, ionizing radiation, X-ray, MCF-7 cells, breast cancer

## Abstract

Radiation therapy, which applies high-energy rays, to eradicate tumor cells, is considered an essential therapy for the patients with breast cancer. Most tumor cells secrete exosomes, which are involved in cell-to-cell communication in tumor tissue and contribute therapeutic resistance and promote tumor aggressiveness. Here, we investigated the effect of clinically applicable doses of X-ray irradiation (2, 4, 6, 8, 10 Gy) on the dynamics of the exosomes’ activity in MCF-7 breast cancer cells. Survival and apoptosis rate of cells against X-ray doses was examined using MTT and flow cytometry assays, respectively. Whereas, the levels of reactive oxygen species (ROS) in the X-ray-treated cells were detected by fluorometric method. The mRNA levels of vital genes involved in exosome biogenesis and secretion including Alix, Rab11, Rab27a, Rab27b, TSPA8, and CD63 were measured by real-time PCR. The protein level of CD63 was examined by Western blotting. Additionally, exosomes were characterized by monitoring acetylcholinesterase activity, transmission electron microscopy, size determination, and zeta potential. The result showed that in comparison with control group cell survival and the percentage of apoptotic cells as well as amount of ROS dose-dependently decreased and increased in irradiated cells respectively (*p* < 0.05). The expression level of genes including Alix, Rab27a, Rab27b, TSPA8, and CD63 as well as the protein level of CD63 upraised according to an increase in X-ray dose (*p* < 0.05). We found that concurrent with an increasing dose of X-ray, the acetylcholinesterase activity, size, and zeta-potential values of exosomes from irradiated cells increased (*p* < 0.05). Data suggest X-ray could activate exosome biogenesis and secretion in MCF-7 cells in a dose-dependent way, suggesting the therapeutic response of cells via ROS and exosome activity.

## 1. Introduction

Breast cancer is the most frequently detected malignancy among women worldwide, with the highest incidence rates in developed countries [1]. It is reported that about 2.1 million new breast cancer cases were diagnosed in 2018 [2]. Breast cancer is the second driving reason for cancer-based mortality among women and the most generally identified cancer after skin-related cancers [3]. Radiation therapy, so called radiotherapy, is among the most important methods of cancer treatment, which applies ionizing radiation (IR) to eradicate cancerous cells [4]. The radiotherapy not only is considered as primary therapy, but also is used along with chemotherapy, hormone therapy, and surgery [5,6].

Exosomes are extracellular vesicles (EVs) released from various cell types under normal and pathological conditions [7]. By transferring a variety of biological compounds such as miRNAs, mRNAs, DNA, proteins, and also lipids, EVs play key roles in cell to cell communication [8]. At the subcellular level, exosomes (30–120 nm) are originated from the endosomes and multivesicular body (MVB) compartments of cells [7,9]. When MVBs containing exosomes fuse with the plasma membrane, exosomes are secreted to the extracellular milieu. Once secreted, exosomes can interact with neighboring cells and affect the activities of recipient cells. As exosomes are distributed through bio-fluids, for example cerebrospinal fluid (CSF), blood, semen, urine, bronchoalveolar lavage, breast milk, bile, and saliva, they can target distantly located recipient cells [7,10]. Increasing evidence indicates that different molecules such as the endosomal sorting complex for transport required (ESCRT) machinery or related components including Alix, tetraspanins (e.g., CD63), and Rab-GTPases are involved in exosome-sorting, trafficking, and combination with the plasma membrane. Various Rab-GTPases control intracellular trafficking of vesicles [11,12]. The roles of Rab27a, Rab27b, and Rab11 in the exosomal secretory pathway have already been confirmed [13,14]. Although different mechanisms have been reported to participate in exosome biogenesis and secretion upon cells reacts to exogenous stimuli, nevertheless, our information is still inadequate [15].

In tumor microenvironment, tumor cells communicate with each other and neighboring stromal cells via exosomes and share biological signals and components. In this regard, tumoral exosomes participate to enhance tumorgenesis, incursion, angiogenesis, immunosuppression, and development of metastatic niche, and may elicit resistance against therapies [16,17]. It has been suggested that stress conditions including reactive oxygen species (ROS), DNA disruption, and impairment of cell organelles are a consequence of IR-induced harmful effects on irradiated cells [18]. Nevertheless, radioresistance, and local relapse accompanied by metastasis usually become a challenge after treatment [19].

It is well-established that IR-irradiated cells can produce soluble factors such as exosomes to affect non-irradiated neighboring cells, which are recognized as a non-targeting effect of IR [20]. Exosomes derived from irradiated cells disseminate IR-induced effects to the non-irradiated cell, and may induce functional responses in recipient cells [21]. Furthermore, exosomes from irradiated cells are capable of causing genetic instability and tumor aggressiveness in non-irradiated cancer cells, and may induce relapse [22]. Mutschelknaus et al. reported that IR (γ-ray) amplified the exosome secretion rate in irradiated BHY tumor cells, a squamous head and neck cancer cell line, and also exosomes from γ-rayed cells stimulated both radioresistance and proliferation responses in non-irradiated BHY cells in vitro [23]. This indicates that IR could alter the kinetics of exosomes secretion pathway in irradiated cancer cells. To our knowledge, there exists a limited number of reports about the kinetics of exosomes secretion and abscission pathway under the IR conditions. Considering that exosomes are the mediators in tumor progression, and that they mediate the responses against chemo- and radiotherapies, we investigated the possible effect of clinically applicable doses of X-ray on exosome biogenesis and abscission in MCF-7 breast cancer cell line.

## 2. Results

### 2.1. Ionizing Radiation Reduces the Viability of MCF-7 Cells

**Figure 1:**
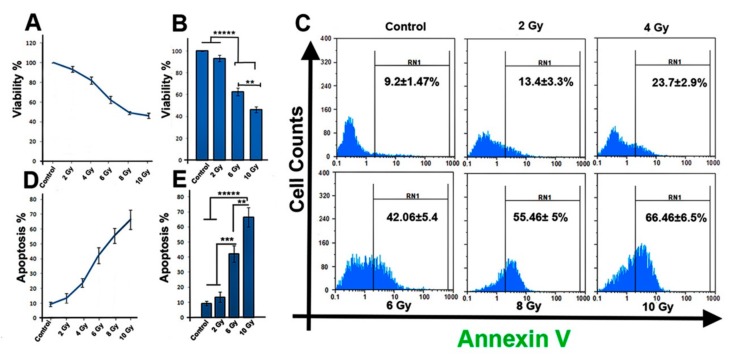
(**A,B**) Ionizing radiation (IR) reduced MCF-7 cells survival rate over 48 h after irradiation. (**C**) Flow cytometric analysis of apoptotic cells in all irradiated groups. (**D**,**E**) Percentage of apoptotic cells was increased in IR-treated cells. One-way ANOVA with Tukey test was applied. All values are means ± SD; *n* = 3. ** p < 0.01, *** p < 0.001, ***** *p* < 0.00001.

MTT assay and a flow cytometry investigation were applied to investigate the effect of IR on cell survival and apoptosis rate 48 h post-exposure. Our screening data showed that the cell viability, apoptosis rate, as well as the ROS production were affected in a dose-dependent manner. We observed significant changes against three doses of irradiation (2, 6, and 10 Gy). As shown in Figure 1A,B, compared to control group, the cell viability was reduced significantly in response to IR after 48 h, indicating dose-dependent reduction at 6 Gy and 10 Gy (*p* < 0.05). However, a minor but not substantial reduction (*p* > 0.05) in cell viability was observed against 2 Gy group versus control group. Compared to control and 2 Gy groups, the 6 Gy and 10 Gy groups exhibited a significant reduction in cell viability (*p* < 0.00001; Figure 1B). Additionally, when 6 Gy and 10 Gy groups were compared, the viability of 10 Gy cells was significantly decreased compared to 6 Gy group (62.56 ± 3.6 vs. 46.4 ± 2.6; *p*_6 Gy vs. 10 Gy group_ < 0.01; Figure 1B). 

### 2.2. Ionizing Radiation Increases the Apoptosis Rate of MCF-7 Cells

The apoptosis rate in MCF-7 cells was also determined 48 h post-exposure. Data from flow cytometry showed that IR induces apoptosis in cells (*p* < 0.05; Figure 1C,D). In comparison to the control group, a significant increase in the Annexin V positive cells population in 10 Gy group was detected in 2 Gy groups (*p*_Control and 2 Gy vs. 10 Gy group_ < 0.00001; Figure 1E). Compared with control and 2 Gy groups, the 6 Gy irradiation caused significantly elevated apoptosis in cells (*p*_Control and 2 Gy vs. 6 Gy group_ < 0.001). Additionally, when 6 Gy and 10 Gy groups were compared, a noticeable increase in the apoptosis rate of cells irradiated with 10 Gy X-ray was observed (42.06 ± 5.4 vs. 66.46 ± 6.5; *p* < 0.01) (Figure 1E). These results indicate that IR could damage MCF-7 cells by inducing apoptosis, and this effect is dose dependent.

### 2.3. Ionizing-Irradiated Cells Exhibit Increased Production of Reactive Oxygen Species

**Figure 2:**
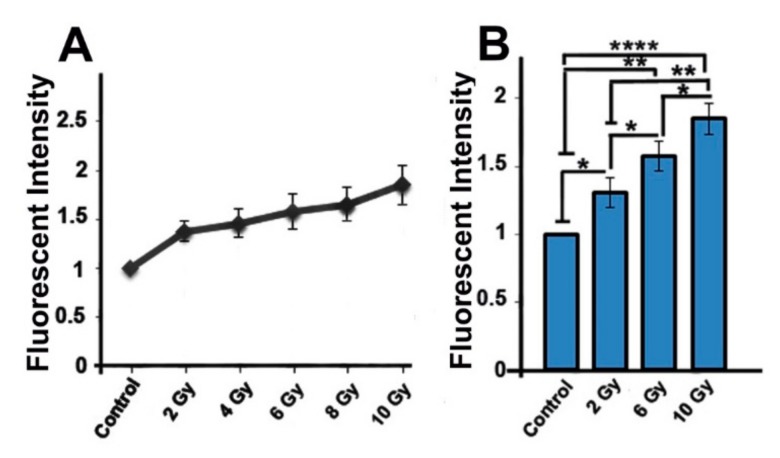
Quantification of reactive oxygen species (ROS) production in all groups (**A**,**B**). One-way ANOVA with Tukey test was applied. All values are means ± SD; *n* = 3. * *p* < 0.05, ** p < 0.01, **** *p* < 0.0001.

In order to observe the oxidative effect of IR on MCF-7 cells, we performed the fluorometric method to assay the ROS generation. Data indicated that exposure of cells to IR resulted in increased ROS production compared to non-irradiated control cells (*p_Control vs.2 Gy group_* < 0.05; *p_Control vs.6 Gy group_* < 0.01; *p_Control_*_vs. *10 Gy group*_ < 0.0001; Figure 2A,B). The level of ROS in 10 Gy group was increased (1.86 ± 0.19) in comparison with 6 Gy (1.57 ± 0.18) and 2 Gy (1.37 ± 0.11) groups (*p* < 0.05, *p* < 0.01, respectively; Figure 2B). The significant increased ROS generation was observed in 6 Gy group in comparison with 2 Gy group (1.57 ± 0.18 vs. 1.37 ± 0.11; *p* < 0.05). This indicates that IR could cause accumulation of ROS in the irradiated cells.

### 2.4. Ionizing Radiation Enhances the Expression of Genes Involved in Exosome Biogenesis/Secretion

**Figure 3:**
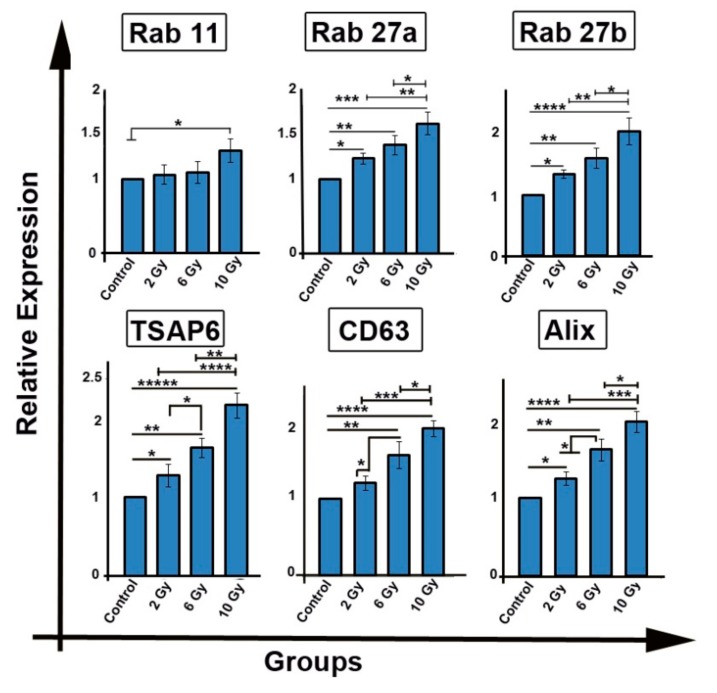
The mRNA levels of genes involved in exosome biogenesis and secretion including Rab11, Rab27a, Rab27b, TSPA6, CD63, and Alix was investigated by qPCR. One-way ANOVA with Tukey test was applied. All values are means ± SD; *n* = 3. * *p* < 0.05, ** p < 0.01, *** p < 0.001, **** *p* < 0.0001, ***** *p* < 0.00001.

Cancer cells exhibit therapeutic resistance, and it has been thought that cancer cells deploy exosomes as conveyers of therapeutic resistance. To observe the influence of IR on exosome production the mRNA levels of genes (Rab 11, Rab 27a, Rab27b, TSAP6, CD63, and Alix transcripts) related to exosome biogenesis and secretion was performed. In comparison with the control group, mRNA level of Rab11 in 10 Gy group was significantly increased (1.39-fold; *p* < 0.05; Figure 3). However, in other groups (2 Gy and 6 Gy), in spite of an increase in Rab11 mRNA transcript in irradiated groups, no significant changes were observed (*p* > 0.05).

Additionally, we found that IR up-regulated the expression of Rab27a gene in treated groups (*p_Control_*_vs. *2 Gy*_*_group_* < 0.05; *p_Control_*_vs. *6 Gy*_*_group_* < 0.01; *p_Control_*_vs. *10 Gy group*_ < 0.001). Compared with either 2 Gy or 6 Gy groups, IR increased the mRNA level of Rab27a in 10 Gy group (*p_2Gy_*_vs. *10 Gy*_*_group_* < 0.01; *p_6 Gy_*_vs. *10 Gy*_*_group_* < 0.05). Similarly, IR amplified the expression of Rab27b gene in irradiated cells (*p_Control vs.2 Gy group_* < 0.05; *p_Control_*_vs. *6 Gy*_*_group_* < 0.01; *p_Control_*_vs. *10 Gy group*_ < 0.0001). Compared to 2 Gy and 6 Gy groups, an increased level of Rab27b transcript was observed in 10 Gy group (*p_2Gy vs.10 Gy group_* < 0.01; *p_6 Gy_*_vs. *10 Gy*_*_group_* < 0.05). 

We also observed that IR induced an elevated expression of TSAP6 in irradiated cells, as compared to control group (*p_Control_*_vs. *2 Gy*_*_group_* < 0.05; *p_Control_*_vs. *6 Gy*_*_group_*< 0.01; *p_Control_*_vs. *10 Gy group*_ < 0.00001; Figure 3). Notably, the transcript level of TSAP6 increased 2.17 ± 0.16-fold in 10 Gy group compared with both 6 Gy (1.63 ± 0.12) and 2 Gy (1.28 ± 0.14) groups (*p* < 0.001, *p* < 0.0001, respectively). Furthermore, 2 Gy and 6 Gy also exhibited significant difference between the mRNA level of TSAP6 (*p* < 0.05).

As shown in Figure 3, upon irradiation of MCF-7 cells, a significant increase in the mRNAs levels of CD63 (*p_Control_*_vs. *6 Gy*_*_group_* < 0.001; *p_Control_*_vs. *10 Gy group*_ < 0.0001; Figure 3) and Alix (*p_Control_*_vs. *2 Gy*_*_group_* < 0.05; *p_Control_*_vs. *6 Gy*_*_group_* < 0.01; *p_Control_*_vs. *10 Gy group*_ < 0.0001) were detected. In comparison with 2 Gy and 6 Gy cells, the expression of CD63 and Alix genes was significantly augmented in 10 Gy irradiated cells (*p_2 Gy_*_vs. *10 Gy*_*_group_* < 0.001; *p_6 Gy_*_vs. *10 Gy group*_ < 0.05). Compared to 2 Gy groups, the expression of CD63 (1.56 ± 0.17) and Alix (1.62 ± 0.14) genes was significantly increased in 6 Gy groups (*p* < 0.05). These results indicate that cancer cells could respond to radiotherapy by producing exosomes and their secretion to extracellular environment. The effect is in a dose-dependent manner.

### 2.5. Acetyl Cholinesterase Activity Was Increased in Ionizing-Radiated MCF-7 Cells

**Figure 4:**
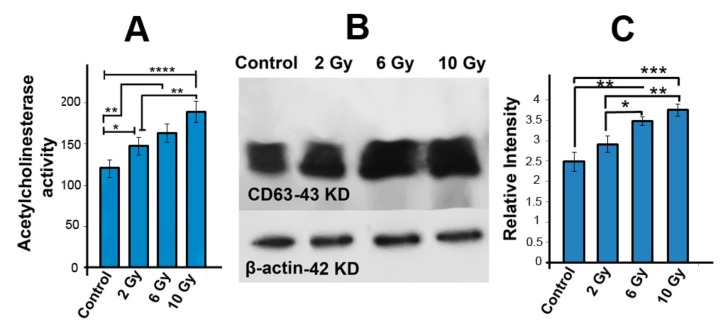
AChE activity assay was examined to measure exosome secretion (**A**). Western blot of CD63 protein in cells (**B**,**C**). Normalization was performed to β-actin. One-way ANOVA with Tukey test was applied. All values are shown as means ± SD; *n* = 3. * *p* < 0.05, ** p < 0.01, *** p < 0.001, **** *p* < 0.0001.

To measure the possible effect of IR on the exosome secretion rate, we used AChE activity assay 48 h post-irradiation. We found that the AChE activity in irradiated groups was enhanced as compared to control group (*p_Control_*_vs. *2 Gy*_*_group_*< 0.05; *p_Control_*_vs. *6 Gy*_*_group_* < 0.01; *p_Control_*_vs. *10 Gy group*_ < 0.0001; Figure 4A). An increased level of AChE activity in 10 Gy group was observed as compared to 2 Gy group (147.65 ± 11.14 vs. 189 ± 12.65; *p* < 0.01; Figure 4A).

### 2.6. The Protein Level of CD63 Was Increased in Irradiated MCF-7 Cells

To study the influence of IR on the protein level of CD63 (an exosome marker), we performed Western blotting assay using the *SDS*-*PAGE* method. Consistent with expression of CD63 at mRNA level in cells, the elevated expression of CD63 at protein level was also observed in exosomes secreted from those cells. Our data show that the protein level of CD63 in 6 Gy (3.48-fold ± 0.11) and 10 Gy (3.75 ± 0.15) increased as compared to control group (2.48 ± 0.18; *p_Control_*_vs. *6 Gy*_*_group_* < 0.01; *p_Control_*_vs. *10 Gy group*_ < 0.001; Figure 4B,C). In addition, when 6 Gy and 10 Gy groups were compared to the 2 Gy group, the protein level of CD63 was increased in 6 Gy and 10 Gy groups (*p_2 Gy_*_vs. *6 Gy*_*_group_* < 0.05; *p_2 Gy_*_vs. *10 Gy group*_ < 0.01; Figure 4B,C), indicating a dose-dependent effect. 

### 2.7. Confirmation of MCF-7 Derived Exosomes by Flow Cytometry and TEM

**Figure 5:**
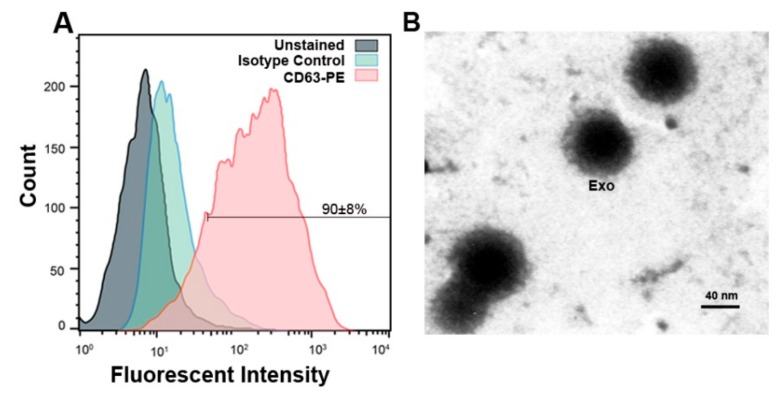
(**A**) Flow cytometric data confirmed exosomes by identifying exosomal marker CD63 and 90 ± 8% of exosomes in suspension represented CD63 marker. (**B**) Representative micrograph from transmission electron microscopy showing the nano-sized exosomes (Scale bar 40 nm). Exo: exosomes.

We used flow cytometry and TEM to characterize MCF-7 cell-derived exosomes. The flow cytometry analysis showed that 90 ± 8% of purified exosomes express exosomal marker CD63 (Figure 5A). Images from TEM indicated that purified exosomes are nano-sized and pose a round shape (Figure 5B). 

### 2.8. IR Alteres the Exosomes Size and Zeta-Potential 

**Figure 6:**
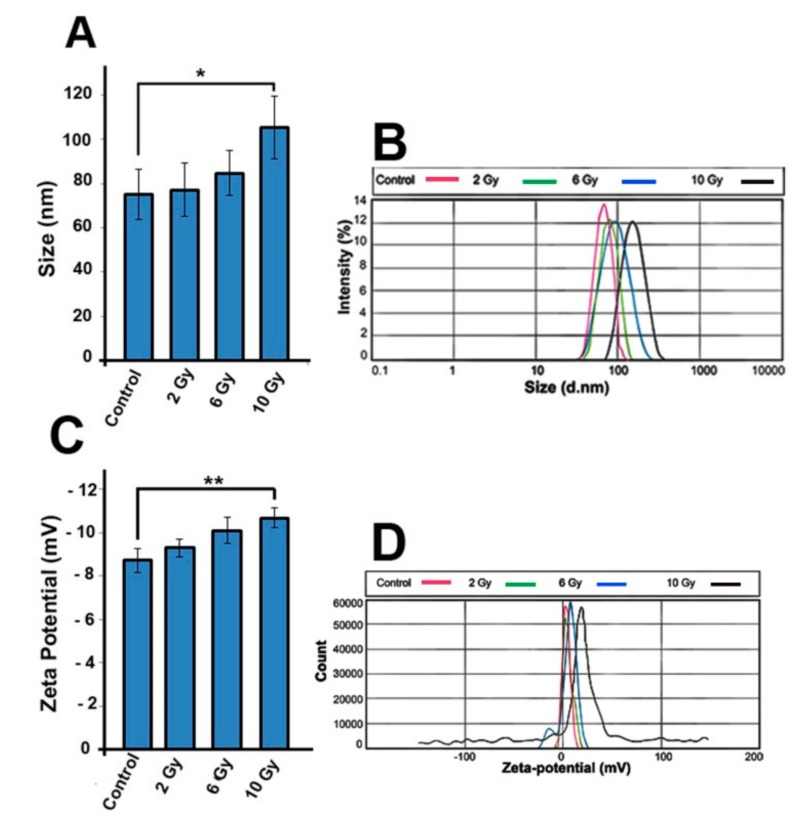
(**A,B**) Size distribution of IR exosomes and (**C**,**D**) zeta potential of purified exosomes were analyzed by dynamic light scattering (DLS). One-way ANOVA with Tukey test was applied. All values are shown as means ± SD; *n* = 3. *p < 0.05, ** p < 0.01.

To evaluate any change in the size and zeta-potential of exosomes from MCF-7 cells, we used dynamic light scattering (DLS) analysis. Our result revealed that IR exposure resulted in a significant increase in exosomes size (Figure 6A,B). Compared to control group, size of exosome was significantly increased in 10 Gy group (75 ± 11.5 vs. −103.34 ± 16.2; *p_Control and 2 Gy_*_vs. *10 Gy group*_ < 0.05; Figure 6). Additionally, the zeta-potential value of 10 Gy exosomes was significantly increased when compared to control exosomes (−8.73 ± 0.5 vs.-10.67 ± 0.46; *p_Control and 2 Gy_*_vs. *10 Gy group*_ < 0.01; Figure 6). These data demonstrate that IR influences the size and zeta-potential of exosomes. 

## 3. Discussion

Despite being the first choice to eradicate tumor cells, radiotherapy represents clinical concerns about resistance to radiotherapy in cancer patients [24,25,26]. Recent studies have shown that although IR inhibits tumor growth, it could, nevertheless, potentially induce bystander effects to non-irradiated tumor cells leading to tumor relapse and metastasis [24,27,28]. The investigation of IR-mediated intercellular communication is important in cancer therapy because the irradiated cancer cells could send various soluble signals such as exosomes to recipient neighboring normal and malignant cells and may subsequently alter function, fate, and proliferation of recipient cells [20,29]. According to previous studies, environmental stimuli and stress conditions such as IR had great impacts on the exosome secretion rate and cargo [22,30]. The characteristics of mechanisms that governed the exosomal secretory pathway were not clearly discovered in clinically applicable doses of X-ray condition.

In this study, based on IR dose intensity, we showed that cell viability rate was reduced. To improve understanding, it is crucial to recognize the causal mechanism of IR-induced cell death. Our results demonstrated that IR induced apoptosis in tumor cells, which were in a good agreement with previous studies [31,32]. Moreover, we showed that the rate of apoptosis was increased according to increase in IR dose. Gudkov and Komarova reported that IR generated DNA single-strand breaks (SSBs) and double-strand breaks (DSBs) and activated downstream molecules involved in intrinsic apoptotic pathway, which collectively led to cancer cell apoptosis [33]. Besides the intrinsic apoptotic pathway, it was reported that IR participates in promoting apoptosis through extrinsic apoptotic pathway, which involved p53/CD95/Fas signaling [34]. We also found that ROS level increased in cells exposed to IR in a dose-dependent manner. Consistent with this, Yamamory et al. demonstrated that IR induced ROS generation in human lung carcinoma A549 cells [35].

The previous report has shown that the reception of IR by irradiated cells could potentially interrupt atomic structures in biological molecules and radiolysis of water was also occurred; by this means, the produced ROS can harm nucleic acids, proteins, and lipids, and consequently may cause cell death [36]. A work by Jelonek and co-workers revealed that the composition of exosomes derived from 2 Gy-treated head and neck squamous cell carcinoma was different from those of non-irradiated cancer cells [37]. To the best of our knowledge, there is a small number of studies that confirmed the IR-induced alterations in the dynamic of exosomes secretion pathway and their key roles in IR-induced intercellular communication [37,38], therefore, we seek to understand how IR affects exosome biogenesis and secretion in MCF-7 cancer cells. The transcript level of genes involved in exosome biogenesis and secretion including Rab11, Rab27a, Rab27b, TSPA6, CD63, and Alix [38] concurrently with CD63 protein level increased in irradiated cells. In this context, we assayed TSAP6 expression, an IR-sensitive protein, and found that the expression of TSAP6 simultaneously increased with other exosomal genes. It was previously demonstrated that IR-induced DNA damage and activated p53 protein, which contributed to inducing TSAP6 activity, in turn, TSAP6 promoted exosome biogenesis [39]. 

Based on our observation, the increased exosomes secretion rate had dose-dependently concurred with an enhanced level of TSAP6 transcript. Compared to other genes, the mRNA level of Rab11 slightly enhanced in irradiated groups, and data showed a significant increase only in 10 Gy cells. Rab11 controls the recovering of membranous structures from the endosomal organizations to the plasma membrane [40] and have been reported to promote exosome biogenesis in the K562 cells in a Ca^2+^-dependent manner [13]. This fact could be explained that the function of Rab11 may depend on type of cancer cells, dose, and time of irradiation. Additionally, we found that activity rate of acetyl cholinesterase, an exosome-associated enzyme, was increased in conditioned media of X-rayed cells, indicating enhanced exosome secretion rate. Similarly, Jella and co-workers found that γ-ray enhanced exosomes secretion rate in human keratinocyte cells in vitro [29]. These results confirmed that the exosome secretion pathway was activated by X-ray 48 h post-irradiation. 

According to a growing body of evidence, the increased activity of exosomal secretory pathway is a canal to expel the damaged molecules in such stress conditions [41,42]. In this regard, Takahashi and co-workers demonstrated that exosome secretion contributes to maintain cell homeostasis by eliminating DNA fragments from cell cytoplasm [43]. Moreover, recent evidence has revealed that exosomes from radiated cells could modify target cell function [44], whereas the IR can alter cargo of exosomes [45]. As previous studies have shown that exosomes from stressed cells induce the stress in unstressed cells, the so-called bystander effect [46]. Similarly, the exosomes secreted from chemo-resistant cells when interacting with nearby cells they induce chemoresistance in recipient cells [47]. Keeping in view such bystander effects, we speculate that in a similar way, that exosomes from IR-treated cells may communicate (induce) resistance to untreated cells.

Furthermore, we aimed to understand whether cell responses to IR by releasing various distinct exosome populations. Our finding showed that exosomes size along with zeta-potential value was increased in the high dose of X-ray (10 Gy), indicating the alteration in the exosome sorting pathway. To our knowledge, these results are preliminary facts and there is little information about the impact of IR on size and zeta-potential of exosomes. However, in our recent work we found that the size of exosomes from diabetic mesenchymal stem cells was increased [48]. Recently, a work by Bagheri and colleagues reported that the zeta-potential of exosomes from endothelial cells irradiated by low-level laser was reduced in a dose-dependent manner but exosomes size was not affected [49]. It was confirmed that exosomes were heterogeneously generated from MVB compartment [50] and the distinct subpopulation of exosomes exhibit different cargo and composition [51]. In this regard, we would hypothesize that IR may alter MVB loading pathways which, in turn, reflected in exosomes cargo [37] and also in size. Nanoparticles such as exosomes with small zeta-potential values have a strong tendency to aggregation. Therefore, particles with high zeta-potential values are less prone to aggregate and are more stable in a distinct solution [52]. Using low laser irradiation on human RBCs, Musavi and co-workers have uncovered a relationship between decrease of zeta-potential value and increased RBCs sedimentation [53]. Moreover, in the case of zeta-potential-mediated physical interaction, exosome–exosome interaction may inhibit exosome delivery pathways due to deactivation of some ligands or/and receptors located on exosomes. In contrast, this interaction may amplify exosome mediated signaling in target cells [54]. Sugar chains including sialic acids located at the exosome membrane are responsible for zeta-potential [55]. We propose that IR altered cell physiology, and any changes in sugar component of the plasma membrane of parental cells may be inherited in the exosome membrane and reflected in exosomes zeta-potential value [55], which in our case, was effected by the dose-dependent increase of IR exposure. Through investigation of irradiated MCF-7 cells with different doses of X-ray, we shed light onto the underlined mechanisms of exosome biogenesis inside MCF-7 cells. We showed that upon increase in MCF-7 cells damage, exosome secretion and activity of exosome secretion pathway enhanced in a dose-dependent manner. The evidence from this study suggests a possible correlation between exosome secretion rate and the cell cytotoxicity indicators including cell survival, apoptosis, and ROS generation. Based on our knowledge, this is preliminary data about the ROS/apoptosis/exosome axis, however the signaling crosslink between exosome secretion and ROS generation and apoptosis pathway still remain elusive. Therefore, additional studies are essential to elucidate that whether increased exosome secretion rate is a mechanism by which cells remove harmfully intracellular molecules or helps tumor mass to escape from IR-induced shrinkage by inducing malignancy/metastasis out of irradiated volume, or it is a way to secrete exosomes against irradiation in order to adjust therapeutic response and reinforce the therapeutic resistance.

## 4. Materials and Methods

### 4.1. Ethical Issue

The ethics committee of Urmia University of Medical Sciences approved the all procedures of this study (ethical approval no: IR.UMSU.REC.1397.211; approved on 27 August 2018).

### 4.2. Cell Culture 

MCF-7 human breast cell line (Pasteur Institute, Tehran, Iran) were cultured in DMEM (Dulbecco’s modified Eagle’s medium, Gibco, Dublin, Ireland) medium containing 10% fetal bovine serum (FBS, Gibco) and 1% penicillin streptomycin (Gibco). The cells were kept in a qualified air humidity of 95% at 37 °C. Subculture was usually done when cells reached 80% confluence by 0.25% trypsin (Sigma Aldrich, Darmstadt, Germany). After 3–6 passages, cells were used for downstream experiments. 

### 4.3. Irradiation Procedure

A clinical 6 MV X-ray beam of a medical linear accelerator system (Siemens AG, Munich, Germany) was used for irradiation. A 1.5 cm thick build-up of polystyrene was used, where the cell monolayers in the plates were positioned at isocenter (dmax = 1.6 cm) and irradiation was done at an output rate of 200 MU/min and a gantry angle of 180°. The MU values were calculated by considering the dose rate of machine being 1 cGy per monitor unit. MCF-7 cells were divided into 6 groups and exposed to 0 (control), 2, 4, 6, 8, and 10 Gy of X-ray beam. Non-irradiated control cells were kept in the same condition that the X-ray-treated cells experienced. After irradiation, the cells were handed to an incubator and maintained for 48 h post-irradiation, prior to evaluation. All experiments were repeated in three independent series of experiments.

### 4.4. Cell Survival Assay

Cell viability of irradiated cells was measured using MTT assay. Briefly, 1 × 10^4^ cells were cultivated in a 96-well plate for 24 h and then exposed to irradiation. After 48 h post irradiation, MTT reagent (Sigma, Darmstadt, Germany) with a 5 mg/mL concentration was poured into each well and the cells were maintained at the 37 °C incubator for 4 h. Next, DMSO (dimethyl sulfoxide) compound was added to liquefy formazan crystals, and absorbencies were read at 570 nm using a microplate reader system (BioTek, Winooski, VT, USA).

### 4.5. Apoptosis Test

48 h after irradiation, the apoptosis rate was monitored in MCF-7 cells using Annexin V kit (eBioscience) according to manufacture recommendations. Briefly, 200 µL of binding buffer was added to cell suspension and kept at room temperature for 15 min. Then, Annexin V-FITC (1 µL/mL) was poured into each sample reaction; and following centrifuge at 1000× *g* for 5 min, 100 µL of binding buffer was mixed with each sample. The percentage of Annexin V positive cells was analyzed using BD FACSCalibur instrument and FlowJo software (version 7.6.1).

### 4.6. Determination of ROS Production

To investigate ROS production in all 6 groups, we performed chemical fluorometric method using ROS assay Kit (E-BC-K138, Elabscience, Wuhan, China). In brief, 1 h after irradiation, 10 μM of dichlorofluorescein diacetate (DCFH-DA) reagent was added to cell culture media and kept at 37 °C for 30 min. Cells were pelleted down through centrifuging at 1000 *g* for 5 min. Following PBS washing twice, the fluorescence was calculated using a plate reader (Bio-Rad, Hercules, CA, USA) at the 485 and 525 nm wavelengths.

### 4.7. Quantitative Real-Time PCR Analysis of Exosome Biogenesis Related Genes

Quantitative real-time PCR analysis was used for the quantification of mRNA levels of Rab11, Rab27a, Rab27b, TSPA6, CD63, and Alix genes involved in the exosome biogenesis and secretion pathway. Total RNA was isolated from different groups of irradiated cells, using RNA isolation kit (YT9080, Yekta Tajhiz Azma, Tehran, Iran) according to manufactures protocol. Following quantification of total RNA concentration by Nanodrop spectrophotometer (BioTek), the RNA was converted into cDNA using cDNA synthesis kit (YT4500, Yekta Tajhiz Azma, Iran). The mRNA levels of following genes Rab11, Rab27a, Rab27b, TSAP6, CD63, and Alix were measured by Q-PCR system (Applied Biosystems) using a SYBR Green dye-based PCR Master Mix (YT2551, Yekta Tajhiz Azma, Iran). Results were normalized to the housekeeping GAPDH gene and quantitative calculations were made by applying formula 2**^−ΔΔ*C*T^** method. The list of the used primers is presented in Table 1.

### 4.8. Western Blotting Analysis

Lysates of irradiated cells were collected using lysis compound (NaCl, Tris–HCl, NP-40 supplemented with protease inhibitor cocktails) for 1 h on ice. Samples were centrifuged at 14,000 rpm for 20 min. Then, the supernatants of all groups were collected, and the total protein concentration was determined applying the Nanodrop system (BioTek). Immunoblots were performed as described previously [48]. Briefly, 100 µg of total proteins was loaded into each well of SDS-gel electrophoresis and then transferred to PVDF membrane (Polyvinylidene Difluoride; 249 Millipore, Burlington, VT, USA). Next, the PVDF membrane was exposed to primary antibody against human CD63 (ab118307, Abcam) according to manufacturer’s recommendation. HRP-conjugated goat-anti-rabbit IgG antibody (Cell Signaling) was added to samples and kept for 1 h at room temperature. Using ECL system, the relative intensity of immunospecific bands were analyzed via ImageJ software ver.1.44p. β-actin protein (Cell Signaling) was used as endogenous control for western blot normalization.

### 4.9. Acetylcholinesterase Assay 

In order to measure the number of exosomes in conditioned media (CM) of irradiated groups, the AhCE activity was examined using a commercial cholinesterase kit (Cat No. BXC080; Biorexfars, Karaj, Iran) according to manufacturer’s procedure. Briefly, after irradiation, MCF-7 cells were carefully washed with PBS and cultivated with FBS-free DMEM for 48 h. CMs were centrifuged at 15,000 *g* for 20 min at 4 °C. The reagent 1 (potassium hexacyanoferrate plus pyrophosphate) was mixed with CMs and incubated for 5 min at room temperature. Following the mixing with 2-butyrylthio-n,n,n-trimethylethanaminium iodide, the absorbance values were recorded at 405 nm at 3 different rest intervals by a microplate reader system (BioTek). AChE activity was calculated by applying the recommended formula: Activity (U/l) = 65,800 × ΔAbs/min.

### 4.10. Exosome Purification

After the irradiation, cells were incubated in FBS free medium, and the supernatants of cells were collected after 48 h of incubation period. The exosomes were isolated from the supernatants by commercially available Exo-spin™ set (EX01-8, Cell Guidance Systems) using manufacturer’s guidance. Cells and cell debris were removed following centrifugation at 300× *g* for 10 min and at 16,000× *g* for 30 min, respectively. Next, 1 mL of Exo-spin™ buffer was mixed with 2 mL of samples and kept overnight at the 4 °C refrigerator. Following centrifugation at 16,000× *g* for 1 h, exosome pellets were obtained and resuspend in 100 µL of PBS (each pellet) for downstream experiments. 

### 4.11. Confirmation of Exosomes

For exosomes characterization, the exosome samples were subjected to transmission electron microscopy (TEM) and flow cytometry analysis. In TEM procedure, exosome suspension samples were fixed with glutaraldehyde 1%, and then loaded on the carbon grids at room temperature. After drying process, grids were washed twice and stained with uranyl acetate 1% for 10 min at room temperature. Exosomes were imaged using TEM system (Philips BioTwin, CM100, Amsterdam The Netherlands) at 80 kV as described previously [56]. In flow cytometry analysis, primary anti-CD63 antibody (ab118307, Abcam) was added to 100 μL of each exosome samples and incubated at the 4 °C refrigerator for 2 h. Afterwards, CD63-PE secondary antibody (12-0639-42, eBioscience, San Diego, CA, USA) was added and maintained at 37 °C for 1 h. Data were evaluated using BD FACSCalibur instrument and FlowJo software (version 7.6.1).

### 4.12. Evaluation the Size and Zeta Potential of Exosomes 

Dynamic light scattering (DLS) analysis and Zetasizer Nano Z system (Nano ZS ZEN 3600, Malvern Panalytical Ltd, Malvern, UK) were used to monitor any change in the size and zeta potential of exosomes, respectively. For this purpose, each suspension containing 25 μL of exosome sample was mixed with a final volume of 1 mL PBS and injected to system with a laser set at 633 nm wavelength at 25 °C. The intensity of the scattered light was monitored at 173°. Data analysis was achieved by Zetasizer software ver. 6.0 

### 4.13. Statistical Analysis

Data indicate the mean of three independent biological experiments ± SD. In all experiments, the statistical significance was evaluated by one-way analysis of variance (ANOVA) and Tukey post hoc test using SPSS software ver. 25. The *p* value *p* < 0.05 was counted as statistically significant. In figures, asterisks show significant difference between groups as * *p* < 0.05, ** *p* < 0.01, *** *p* < 0.001, **** *p* < 0.0001, and ***** *p* < 0.00001. 

## 5. Conclusions

Taken together, our results demonstrate that various doses of X-ray irradiation (2, 6, 10 Gy) havesubstantial effects on the activated biogenesis and secretion of exosomes in MCF-7 breast cancer cells in a dose-dependent relationship. Our findings indicated that X-ray irradiation could stimulate therapeutic response of cells via ROS and exosome activity. Further studies are essential to uncover the key role and mechanisms of IR-induced overactivation in the dynamic of exosome activities, and better understanding on therapeutic resistance mediated by exosomes to improve outcome of radiotherapy.

## Figures and Tables

**Table 1:** Primers list.

Genes	Sequence (5′→3′)	Tm
*Rab11*	**F:** CCTCAGCCTCTACGAAGCAAA **R:**CCGGAAGTTGATCTCCTCCTG	59
*Rab27a*	**F:** AGAGGAGGAAGCCATAGCAC **R:** CATGACCATTTGATCGCACCAC	59
*Rab27b*	**F:** GGAACTGGCTGACAAATATGG **R:** CAGTATCAGGGATTTGTGTCTT	59
*TSAP6*	**F:** CCTCTACAGCTTCTGCTTGCC **R:** TAGATCTCCATCCGCCAGACC	63
*CD63*	**F:** TCCTGAGTCAGACCATAATCC **R:** GATGGCAAACGTGATCATAAG	63
*Alix*	**F:** CTGGAAGGATGCTTTCGATAAAGG **R:** AGGCTGCACAATTGAACAACAC	63
*GAPDH*	**F:** CAAGTTCAACGGCACAGTCAAG **R:** ATACTCAGCACCAGCATCACC	60

## Abbreviations

ESCRT	Endosomal Sorting Complex required for Transport
DLS	Dynamic Light scattering
DMEM	Dulbecco’s Modified Eagle’s Medium
DMSO	Dimethyl Sulfoxide
DSBs	Double-Strand Breaks
PVDF	Polyvinylidene Difluoride
IR	Ionizing Radiation
Q-PCR	Quantitative real-time PCR
ROS	Reactive Oxygen Species
SSBs	Single-Strand Breaks
TEM	Transmission Electron Microscopy

## References

[1] Ferlay J., Soerjomataram I., Dikshit R., Eser S., Mathers C., Rebelo M., Parkin D.M., Forman D., Bray F. (2015). Cancer incidence and mortality worldwide: Sources, methods and major patterns in globocan 2012. Int. J. Cancer.

[2] Bray F., Ferlay J., Soerjomataram I., Siegel R.L., Torre L.A., Jemal A. (2018). Global cancer statistics 2018: Globocan estimates of incidence and mortality worldwide for 36 cancers in 185 countries. CA: A Cancer J. Clin..

[3] Khalkhali H.R., Lotfnezhad Afshar H., Esnaashari O., Jabbari N. (2016). Applying data mining techniques to extract hidden patterns about breast cancer survival in an iranian cohort study. J. Res. Health Sci..

[4] Esmaeili Govarchin Ghaleh H., Zarei L., Mansori Motlagh B., Jabbari N. (2019). Using cuo nanoparticles and hyperthermia in radiotherapy of mcf-7 cell line: Synergistic effect in cancer therapy. Artif. Cells Nanomed. Biotechnol..

[5] Jabbari N., Zarei L., Esmaeili Govarchin Galeh H., Mansori Motlagh B. (2018). Assessment of synergistic effect of combining hyperthermia with irradiation and calcium carbonate nanoparticles on proliferation of human breast adenocarcinoma cell line (mcf-7 cells). Artif. Cells Nanomed. Biotechnol..

[6] Tutt A., Yarnold J. (2006). Radiobiology of breast cancer. Clin. Oncol..

[7] Nawaz M., Camussi G., Valadi H., Nazarenko I., Ekström K., Wang X., Principe S., Shah N., Ashraf N.M., Fatima F. (2014). The emerging role of extracellular vesicles as biomarkers for urogenital cancers. Nat. Rev. Urol..

[8] Nawaz M., Fatima F., Vallabhaneni K.C., Penfornis P., Valadi H., Ekström K., Kholia S., Whitt J.D., Fernandes J.D., Pochampally R. (2016). Extracellular vesicles: Evolving factors in stem cell biology. Stem Cells Int..

[9] Van Niel G., D‘Angelo G., Raposo G. (2018). Shedding light on the cell biology of extracellular vesicles. Nat. Rev. Mol. Cell Biol..

[10] Théry C., Witwer K.W., Aikawa E., Alcaraz M.J., Anderson J.D., Andriantsitohaina R., Antoniou A., Arab T., Archer F., Atkin-Smith G.K. (2018). Minimal information for studies of extracellular vesicles 2018 (misev2018): A position statement of the international society for extracellular vesicles and update of the misev2014 guidelines. J. Extracell. Vesicles.

[11] Hessvik N.P., Llorente A. (2018). Current knowledge on exosome biogenesis and release. Cell. Mol. Life Sci..

[12] Bobrie A., Colombo M., Raposo G., Théry C. (2011). Exosome secretion: Molecular mechanisms and roles in immune responses. Traffic.

[13] Savina A., Fader C.M., Damiani M.T., Colombo M.I. (2005). Rab11 promotes docking and fusion of multivesicular bodies in a calcium-dependent manner. Traffic.

[14] Ostrowski M., Carmo N.B., Krumeich S., Fanget I., Raposo G., Savina A., Moita C.F., Schauer K., Hume A.N., Freitas R.P. (2010). Rab27a and rab27b control different steps of the exosome secretion pathway. Nat. Cell Biol..

[15] Alenquer M., Amorim M. (2015). Exosome biogenesis, regulation, and function in viral infection. Viruses.

[16] Nawaz M., Fatima F., Nazarenko I., Ekström K., Murtaza I., Anees M., Sultan A., Neder L., Camussi G., Valadi H. (2016). Extracellular vesicles in ovarian cancer: Applications to tumor biology, immunotherapy and biomarker discovery. Expert Rev. Proteom..

[17] Fatima F., Nawaz M. (2017). Vesiculated long non-coding rnas: Offshore packages deciphering trans-regulation between cells, cancer progression and resistance to therapies. Non-Coding RNA.

[18] Wang J.-S., Wang H.-J., Qian H.-L. (2018). Biological effects of radiation on cancer cells. Mil. Med. Res..

[19] Neville B.W., Day T.A. (2002). Oral cancer and precancerous lesions. CA: A Cancer J. Clin..

[20] Al-Mayah A., Bright S., Chapman K., Irons S., Luo P., Carter D., Goodwin E., Kadhim M. (2015). The non-targeted effects of radiation are perpetuated by exosomes. Mutat. Res./Fundam. Mol. Mech. Mutagenesis.

[21] Diamond J.M., Vanpouille-Box C., Spada S., Rudqvist N.-P., Chapman J.R., Ueberheide B.M., Pilones K.A., Sarfraz Y., Formenti S.C., Demaria S. (2018). Exosomes shuttle trex1-sensitive ifn-stimulatory dsdna from irradiated cancer cells to dcs. Cancer Immunol. Res..

[22] Arscott W.T., Tandle A.T., Zhao S., Shabason J.E., Gordon I.K., Schlaff C.D., Zhang G., Tofilon P.J., Camphausen K.A. (2013). Ionizing radiation and glioblastoma exosomes: Implications in tumor biology and cell migration. Transl. Oncol..

[23] Mutschelknaus L., Peters C., Winkler K., Yentrapalli R., Heider T., Atkinson M.J., Moertl S. (2016). Exosomes derived from squamous head and neck cancer promote cell survival after ionizing radiation. PLoS ONE.

[24] Ghisolfi L., Keates A.C., Hu X., Lee D.-k., Li C.J. (2012). Ionizing radiation induces stemness in cancer cells. PLoS ONE.

[25] Pickhard A.C., Margraf J., Knopf A., Stark T., Piontek G., Beck C., Boulesteix A.-L., Scherer E.Q., Pigorsch S., Schlegel J. (2011). Inhibition of radiation induced migration of human head and neck squamous cell carcinoma cells by blocking of egf receptor pathways. BMC Cancer.

[26] Zhou Y.-C., Liu J.-Y., Li J., Zhang J., Xu Y.-Q., Zhang H.-W., Qiu L.-B., Ding G.-R., Su X.-M., Guo G.-Z. (2011). Ionizing radiation promotes migration and invasion of cancer cells through transforming growth factor-beta–mediated epithelial–mesenchymal transition. Int. J. Radiat. Oncol. Biol. Phys..

[27] Phillips T.M., McBride W.H., Pajonk F. (2006). The response of cd24−/low/cd44+ breast cancer–initiating cells to radiation. J. Natl. Cancer Inst..

[28] Lee S.Y., Jeong E.K., Ju M.K., Jeon H.M., Kim M.Y., Kim C.H., Park H.G., Han S.I., Kang H.S. (2017). Induction of metastasis, cancer stem cell phenotype, and oncogenic metabolism in cancer cells by ionizing radiation. Mol. Cancer.

[29] Kumar Jella K., Rani S., O‘driscoll L., McClean B., Byrne H., Lyng F. (2014). Exosomes are involved in mediating radiation induced bystander signaling in human keratinocyte cells. Radiat. Res..

[30] De Jong O.G., Verhaar M.C., Chen Y., Vader P., Gremmels H., Posthuma G., Schiffelers R.M., Gucek M., van Balkom B.W. (2012). Cellular stress conditions are reflected in the protein and rna content of endothelial cell-derived exosomes. J. Extracell. Vesicles.

[31] Chin C., Bae J.H., Kim M.J., Hwang J.Y., Kim S.J., Yoon M.S., Lee M.K., Kim D.W., Chung B.S., Kang C.D. (2005). Radiosensitization by targeting radioresistance-related genes with protein kinase a inhibitor in radioresistant cancer cells. Exp. Mol. Med..

[32] Meir O., Dvash E., Werman A., Rubinstein M. (2010). C/ebp-β regulates endoplasmic reticulum stress–triggered cell death in mouse and human models. PLoS ONE.

[33] Gudkov A.V., Komarova E.A. (2003). The role of p53 in determining sensitivity to radiotherapy. Nat. Rev. Cancer.

[34] Sheard M.A. (2001). Ionizing radiation as a response-enhancing agent for cd95-mediated apoptosis. Int. J. Cancer.

[35] Yamamori T., Yasui H., Yamazumi M., Wada Y., Nakamura Y., Nakamura H., Inanami O. (2012). Ionizing radiation induces mitochondrial reactive oxygen species production accompanied by upregulation of mitochondrial electron transport chain function and mitochondrial content under control of the cell cycle checkpoint. Free Radic. Biol. Med..

[36] Azzam E.I., Jay-Gerin J.-P., Pain D. (2012). Ionizing radiation-induced metabolic oxidative stress and prolonged cell injury. Cancer Lett..

[37] Jelonek K., Wojakowska A., Marczak L., Muer A., Tinhofer-Keilholz I., Lysek-Gladysinska M., Widlak P., Pietrowska M. (2015). Ionizing radiation affects protein composition of exosomes secreted in vitro from head and neck squamous cell carcinoma. Acta Biochim. Pol..

[38] Jelonek K., Widlak P., Pietrowska M. (2016). The influence of ionizing radiation on exosome composition, secretion and intercellular communication. Protein Pept. Lett..

[39] Lespagnol A., Duflaut D., Beekman C., Blanc L., Fiucci G., Marine J.-C., Vidal M., Amson R., Telerman A. (2008). Exosome secretion, including the DNA damage-induced p53-dependent secretory pathway, is severely compromised in tsap6/steap3-null mice. Cell Death Differ..

[40] Blanc L., Vidal M. (2018). New insights into the function of rab gtpases in the context of exosomal secretion. Small GTPases.

[41] Lehmann B.D., Paine M.S., Brooks A.M., McCubrey J.A., Renegar R.H., Wang R., Terrian D.M. (2008). Senescence-associated exosome release from human prostate cancer cells. Cancer Res..

[42] Pan B.-T., Teng K., Wu C., Adam M., Johnstone R.M. (1985). Electron microscopic evidence for externalization of the transferrin receptor in vesicular form in sheep reticulocytes. J. Cell Biol..

[43] Takahashi A., Okada R., Nagao K., Kawamata Y., Hanyu A., Yoshimoto S., Takasugi M., Watanabe S., Kanemaki M.T., Obuse C. (2017). Exosomes maintain cellular homeostasis by excreting harmful DNA from cells. Nat. Commun..

[44] Jella K., Nasti T., Li Z., Lawson D., Ahmed R., Dynan W., Khan M. (2018). Post-irradiated tumor-derived exosomes lead to melanoma tumor growth delay, potentially mediated by death associated molecular pattern (damps) proteins. Int. J. Radiat. Oncol. Biol. Phys..

[45] Mutschelknaus L., Azimzadeh O., Heider T., Winkler K., Vetter M., Kell R., Tapio S., Merl-Pham J., Huber S.M., Edalat L. (2017). Radiation alters the cargo of exosomes released from squamous head and neck cancer cells to promote migration of recipient cells. Sci. Rep..

[46] Bewicke-Copley F., Mulcahy L.A., Jacobs L.A., Samuel P., Akbar N., Pink R.C., Carter D.R.F. (2017). Extracellular vesicles released following heat stress induce bystander effect in unstressed populations. J. Extracell. Vesicles.

[47] Samuel P., Mulcahy L.A., Furlong F., McCarthy H.O., Brooks S.A., Fabbri M., Pink R.C., Carter D.R.F. (2017). Cisplatin induces the release of extracellular vesicles from ovarian cancer cells that can induce invasiveness and drug resistance in bystander cells. Philos. Trans. R. Soc. B: Biol. Sci..

[48] Rezaie J., Nejati V., Khaksar M., Oryan A., Aghamohamadzadeh N., Shariatzadeh M.A., Rahbarghazi R., Mehranjani M.S. (2018). Diabetic sera disrupted the normal exosome signaling pathway in human mesenchymal stem cells in vitro. Cell Tissue Res..

[49] Bagheri H.S., Mousavi M., Rezabakhsh A., Rezaie J., Rasta S.H., Nourazarian A., Avci Ç.B., Tajalli H., Talebi M., Oryan A. (2018). Low-level laser irradiation at a high power intensity increased human endothelial cell exosome secretion via wnt signaling. Lasers Med Sci..

[50] Edgar J.R., Eden E.R., Futter C.E. (2014). Hrs-and cd63-dependent competing mechanisms make different sized endosomal intraluminal vesicles. Traffic.

[51] Willms E., Johansson H.J., Mäger I., Lee Y., Blomberg K.E.M., Sadik M., Alaarg A., Smith C.E., Lehtiö J., Andaloussi S.E. (2016). Cells release subpopulations of exosomes with distinct molecular and biological properties. Sci. Rep..

[52] Martins T.S., Catita J., Rosa I.M., e Silva O.A.d.C., Henriques A.G. (2018). Exosome isolation from distinct biofluids using precipitation and column-based approaches. PLoS ONE.

[53] Al Musawi M.S., Jaafar M., Al-Gailani B., Ahmed N.M., Suhaimi F.M. (2017). Laser-induced changes of in vitro erythrocyte sedimentation rate. Lasers Med Sci..

[54] Beit-Yannai E., Tabak S., Stamer W.D. (2018). Physical exosome: Exosome interactions. J. Cell. Mol. Med..

[55] Akagi T., Ichiki T. (2016). Evaluation of zeta-potential of individual exosomes secreted from biological cells using a microcapillary electrophoresis chip. Encyclopedia of Biocolloid and Biointerface Science 2V Set.

[56] Van der Pol E., Coumans F., Varga Z., Krumrey M., Nieuwland R. (2013). Innovation in detection of microparticles and exosomes. J. Thromb. Haemost..

